# Looking to the future of zebrafish as a model to understand the genetic basis of eye disease

**DOI:** 10.1007/s00439-019-02055-z

**Published:** 2019-08-17

**Authors:** Florencia Cavodeassi, Stephen W. Wilson

**Affiliations:** 1 Institute of Medical and Biomedical Education, St. George’s, University of London, Cranmer Terrace, London, SW17 0RE UK; 2 Department of Cell and Developmental Biology, Biosciences, UCL, Gower St, London, WC1E 6BT UK

## Abstract

In this brief commentary, we provide some of our thoughts and opinions on the current and future use of zebrafish to model human eye disease, dissect pathological progression and advance in our understanding of the genetic bases of microphthalmia, andophthalmia and coloboma (MAC) in humans. We provide some background on eye formation in fish and conservation and divergence across vertebrates in this process, discuss different approaches for manipulating gene function and speculate on future research areas where we think research using fish may prove to be particularly effective.

## Background

Congenital malformations of the eyes are amongst the main causes of childhood blindness. Microphthalmia (M), anophthalmia (A) and coloboma (C; collectively MAC syndrome with overlapping phenotypes) are the most severe of these malformations, and together account for up to 11% of the cases of childhood blindness (Williamson and FitzPatrick 2014). MAC phenotypes arise from defects during the earliest stages of embryonic eye development. In the last 20 years, advances in our understanding of the molecular mechanisms driving eye development, together with improved genetic diagnosis techniques, have led to the identification of many genes that when disrupted lead to MAC phenotypes in humans. Despite all these advances, our understanding of eye disease progression in humans is still fragmentary, and most of the molecular causes underlying eye malformations are unidentified. Indeed, the genes identified so far explain only around one-third of the reported cases, accounting for only a small subset of the potentially causative genes (Plaisancie et al. 2019; Reis and Semina 2015; Williamson and FitzPatrick 2014).

Work on animal models is central to our ability to dissect eye development in normal and pathological conditions and, in recent years, the zebrafish has emerged as a great model to elucidate human eye disease (Chhetri et al. 2014; Richardson et al. 2017). Zebrafish are easy to maintain in laboratory conditions and produce large clutches of fast developing, transparent embryos, amenable to embryological and genetic manipulations. Over the last two to three decades, extensive collections of fish lines carrying mutations in genes involved in early embryonic development have been produced—some of these mutations in genes important for eye formation and associated with eye malformations in humans. In addition, many transgenic lines have been generated to enable the close monitoring of organogenesis and the manipulation of gene activity in a tissue-directed way. More recently, advances in genome editing technologies have opened the door to targeted mutagenesis and, with this, the possibility to reproduce the specific mutations found in affected patients, further increasing our ability to explore aetiology and pathological progression in disease models.

In this brief commentary, we provide some of our thoughts and opinions on the current and future use of zebrafish to model human eye disease, dissect pathological progression and advance our understanding of the genetic bases of MAC in humans. We do not extensively review work on eye formation in zebrafish as there are many other recent publications that do this very effectively. We provide some background on eye formation in fish and conservation and divergence across vertebrates in this process, discuss different approaches for manipulating gene function and speculate on future research areas where we think research using fish may prove to be particularly effective. For instance, most of the genes associated with eye malformations identified to date lead to defects when disrupted in isolation but in many cases mutations in a single gene may not display any defect unless combined with other genetic mutations or environmental risk factors. Indeed, these combinatorial conditions likely account for the majority of the MAC cases in humans and may also underlie the huge variation in penetrance and expressivity of congenital eye phenotypes. Technological advances in genome editing will allow us start identifying these genetic interactions and the complex networks in which they participate. We envision that the zebrafish will be instrumental for these advances.

## From eye field to optic cup

The possibility of placing fish embryos under a microscope and acquiring long-term movies at a resolution that allows us to follow single cell behaviours makes these animals particularly well suited to dissect eye patterning and morphogenesis. Indeed, due to the accessibility of fish embryos and their amenability to imaging and manipulation, much of what we know on eye formation from a mechanistic point of view has been learned from studying fish models. We will not attempt here to review in detail those studies, since many excellent reviews on many aspects of eye research in fish have been recently published and we refer readers to them (Angueyra and Kindt 2018; Bazin-Lopez et al. 2015; Blanco-Sanchez et al. 2017; Cavodeassi 2018; Chhetri et al. 2014; Fuhrmann 2010; Fuhrmann et al. 2014; Gestri et al. 2012; Giger and Houart 2018; Martinez-Morales et al. 2017; Moreno-Marmol et al. 2018; Niklaus and Neuhauss 2017; Richardson et al. 2017; Sinn and Wittbrodt 2013; Stenkamp 2015; Wan and Goldman 2016; Zheng et al. 2018; and others).

Eye development starts with the specification of the eye field in the anterior portion of the neural plate, the primordium of the vertebrate central nervous system. Eye field cells are defined by their expression of a group of genes collectively known as eye field specification genes (Zuber et al. 2003). This group of transcription factors initiates a morphogenetic and specification program that in the zebrafish leads to the segregation of the eye field cells from those in surrounding neural plate territories (Bielen and Houart 2012; Brown et al. 2010; Cavodeassi et al. 2005; Cavodeassi et al. 2013), and their evagination from the lateral walls of the forming neural tube. As they do so, eye field cells acquire a neuroepithelial organisation and eventually give rise to the optic vesicles (Ivanovitch et al. 2013; Rembold et al. 2006). The optic vesicles subsequently fold over themselves to give rise to the optic cups (Heermann et al. 2015; Icha et al. 2016; Kwan et al. 2012; Picker et al. 2009). In the zebrafish, optic cup folding occurs concomitant to the subdivision of the primordium in neural retina (NR) and retinal pigment epithelium (RPE). Extensive changes in cell organisation and shape accompany optic cup folding (Cavodeassi 2018; Martinez-Morales et al. 2017), and the final result is a hemispheric cup in which the NR is located in the internal layer of the cup, covered by the thin external RPE layer. The optic cup remains connected to the embryonic brain by the optic stalk, which will subsequently differentiate to give rise to the glial cells that ensheath the optic nerve (Macdonald et al. 1997). The folding of the optic vesicle to give rise to the optic cup is asymmetric and as a result a fissure (called the optic or choroid fissure) forms along the ventral portion of the eye primordium and optic stalk (Bazin-Lopez et al. 2015; Patel and Sowden 2019). The optic fissure is a transient structure, which fuses at late stages of eye development to give rise to a continuous NR and RPE (Gestri et al. 2018; James et al. 2016; Knickmeyer et al. 2018; Patel and Sowden 2019).

Morphologically, the eye primordium in zebrafish and mammals show some differences. For example, the cells in the zebrafish eye primordium only organise as a neuroepithelium as they evaginate from the lateral walls of the forming neural tube (Ivanovitch et al. 2013), while in mammals the tissue is already organised as a columnar epithelium prior to evagination (Svoboda and O’Shea 1987). As the optic vesicles form, an expanded lumen is observed in mammals whereas in fish, the two layers of the optic vesicle are tightly apposed to each other on their apical sides. As the fish optic cup forms, the NR becomes a thick and highly proliferating pseudostratified neuroepithelium, while the RPE undergoes an extensive change in shape, becoming a thin squamous epithelium (Martinez-Morales et al. 2017; Moreno-Marmol et al. 2018). In mammals, these morphological differences between NR and RPE are less evident, and while the NR does eventually become a thick neuroepithelium, the RPE maintains a cuboidal appearance throughout early eye morphogenesis (Eiraku et al. 2011; Heavner and Pevny 2012). Moreover, in the fish eye primordium, NR cells reorganise extensively as they relocate to the internal layer of the optic cup in a process known as rim involution (Heermann et al. 2015; Kwan et al. 2012; Picker et al. 2009; Sidhaye and Norden 2017), a morphogenetic event that has not been described in mammals. These morphological differences may impact on the mechanisms driving eye morphogenesis in each model. However, the genetic networks involved in the progression of eye development are remarkably conserved (Beccari et al. 2013; Martinez-Morales 2016), suggesting that, despite these apparent morphological differences, the eye morphogenesis program is largely conserved.

Recent improvements in 3D culture techniques have enabled the growth of mouse and human eye organoids and this has provided us with the possibility to image eye morphogenesis in these mammalian models and compare the process with that described in fish (Eiraku et al. 2012, 2011; Nakano et al. 2012; Sasai et al. 2012). So far, these studies have revealed more similarities than differences in the mechanisms driving eye formation across species. For example, optic vesicle evagination in zebrafish requires the assembly of a Laminin-rich extracellular matrix around the eye primordium (Bryan et al. 2016; Ivanovitch et al. 2013), a prerequisite also essential for mammalian organoid eye evagination (Eiraku et al. 2011; Nakano et al. 2012). Patterning of the optic vesicle in NR and RPE is tightly coupled to optic cup morphogenesis (reviewed in (Moreno-Marmol et al. 2018) and even though the morphology of the RPE is different in fish and mammals, the mechanical properties acquired by this tissue seem to be similar in both models suggesting a common mechanical role driving eye morphogenesis (Carpenter et al. 2015; Eiraku et al. 2012; Okuda et al. 2018). The cellular mechanisms involved in optic fissure closure have recently started to be dissected at a cellular level in the zebrafish (Gestri et al. 2018; James et al. 2016; Knickmeyer et al. 2018), and other organisms (Hardy et al. 2019; Kelberman et al. 2014; Patel and Sowden 2019) although much remains to be resolved. Nevertheless, the morphology and disposition of the tissues around the fissure are identical in fish and other animals, and it is probable that the cellular mechanisms involved in fissure closure will be largely comparable.

The dissection of eye morphogenesis in fish has so far provided us with substantial insights into the cellular mechanisms and molecular modulators involved in this process. Moreover, sophisticated molecular and imaging approaches have been developed, which make the zebrafish a very powerful tool to dissect eye formation and malformation. In the next section, we review some of the approaches and recent technological advances that allow us to manipulate gene function and exploit the advantages of this system to model human eye disease.

## Mutants, morphants or crispants?

Eye development research in zebrafish may have the primary goal of understanding the fundamental genetic, molecular and cellular mechanisms underlying eye formation or may be focussed more explicitly on modelling or understanding specific congenital abnormalities of eye formation in humans. The particular goals of the research are likely to influence the methods chosen to conduct experiments and there are many possible ways to manipulate gene function in developing zebrafish. We would like to comment briefly on the three most widely used approaches to abrogate gene function in zebrafish: stable lines carrying mutations (mutants); morpholino-based knock-down of gene function (morphants); and use of Crispr/Cas9 gene editing to disrupt gene function in injected embryos (so-called crispants).

The zebrafish came to prominence as a laboratory model system in large part due to the ability to do forward genetic screens to identify developmental phenotypes (Granato and Nusslein-Volhard 1996; Haffter et al. 1996). In such screens, mutants are selected for study on the basis of phenotypes of interest and consequently are unbiased with respect to the identity of the affected gene. This contrasts with reverse genetic approaches in which one selects genes of potential interest and then secondarily determines the consequences of disrupting gene function. Forward genetic screens have identified hundreds of mutations giving rise to phenotypes affecting the developing eye, many of which have been instrumental in defining the key signalling pathways and transcription factors underlying eye formation (Richardson et al. 2017). A major advantage of forward genetic approaches is that by starting with an interesting phenotype, one knows that the affected gene must play an important role in the process being studied. By isolating and identifying causative mutations in multiple mutants with similar phenotypes, it is possible to reveal much about the genetic pathways underlying the aspects of eye formation being studied. In fish, as in humans, advances in RNA and genome sequencing technologies have made it much simpler to identify the genetic mutations underlying the phenotypes, but making the phenotype to genotype link can still, on occasion, prove to be very challenging. Although forward genetic screening has made a huge contribution to our understanding of the genetic basis of eye formation, the advent of simple genome editing has, perhaps, lessened the enthusiasm for conducting such screens. However, cleverly designed screens should continue to be performed and will certainly continue to reveal genetic insights into eye formation in ways that would be hard to achieve through other approaches.

In the last few years, genome editing technologies, particularly based on Crispr/Cas9 (Liu et al. 2019; Prykhozhij et al. 2017), have made it relatively simple to generate lines of zebrafish carrying mutations in targeted genes. To date, by far the most commonly used approach in zebrafish has been to generate loss of function alleles through imprecise DNA repair by the non-homologous end-joining pathway. Triggering other DNA repair mechanisms such as homology directed repair is currently much less efficient, although this may well change as methods evolve. Genome editing now renders it relatively straightforward to create mutant alleles in zebrafish orthologues of candidate or proven human eye disease genes.

Although Crispr/Cas9-based methods are revolutionising studies in fish as in other experimental models, genetics is rarely simple and straightforward and many null mutations may lack obvious or expected phenotypic consequences. This is true in all species studies and for instance, the extensive use of whole exome/genome sequencing in human communities has also revealed the existence of human gene “knockouts” that have no obvious phenotypic consequences, indicating that the prevalence of homozygous null mutations in the population is higher than previously suspected (Saleheen et al. 2017; Sulem et al. 2015). There may be many reasons why phentoypes are not expressed including functional redundancy with paralogous gene(s) (Barshir et al. 2018; Hurles 2004; Wagner 1996). Another possibility is that the loss of function of the affected protein is buffered by the robust nature of the functional network in which the protein participates—a process known as distributed robustness (Wagner 2005).

Most recently, a remarkable process called genetic compensation has been discovered (El-Brolosy et al. 2019; Ma et al. 2019; El-Brolosy and Stainier 2017; Rossi et al. 2015). If a mutant allele triggers nonsense-mediated decay, then this can lead to transcriptional adaptation and upregulation of other genes that can functionally compensate for the loss of gene function. This process is independent of the activity of the protein network in which the gene product would normally function. Instead, the trigger for compensation seems to be either the DNA lesion, or the mutant transcript. Although there are a few clues as to how this may happen (El-Brolosy et al. 2019; Ma et al. 2019; Rossi et al. 2015), there is still much to learn about the mechanistic basis of genetic compensation and transcriptional adaptation. It is also not yet clear how prevalent the process is and how important it is in influencing the expression of phenotypes. Potentially it could be a very widespread phenomenon influencing genetic networks and phenotypic penetrance and expressivity both in health and in disease states.

Despite these considerations, genome editing approaches will continue to be a dominant approach in creating zebrafish models of human eye disease. In using this approach, it is advisable to always create at least two alleles and if one wants to definitely avoid genetic compensation, then one should design null alleles that do not trigger nonsense-mediated decay; for instance, mutant alleles that fail to generate a transcript will obviously lack function and will not trigger genetic compensation (El-Brolosy et al. 2019).

Crispr/Cas9 genome editing is so efficient that one can sometimes see loss of function phenotypes in the injected F0 zebrafish embryos (Wu et al. 2018), with such embryos referred to as crispants. This is, of course, a remarkably quick and efficient way to look at the consequences of loss of gene function but is it a reliable and robust method? As yet there are not many publications that have rigorously assessed the effectiveness of this approach but the signs are promising that it will become a useful addition to the toolbox for genetic studies in zebrafish. Building on published studies (Wu et al. 2018), pilot studies in our hands and those of our colleagues (particularly Francois Kroll and Jason Rihel) in which the approach is used for targeting genes with known loss of function phenotypes, we can obtain expected phenotypes in nearly all injected embryos when using three or four guide RNAs. This implies loss of most/all wild-type alleles and this can be verified by sequencing the genome around the target site. So it seems likely that many genes can be effectively targeted in F0-injected fish.

When using multiple guide RNAs, the crispant approach generates mosaicism in that different cells are very likely to have different mutant allele combinations and consequently this is a different situation to fish carrying stably inherited mutant alleles. Although in most cases one might expect most alleles to be loss of function, it is nevertheless hard to predict if and how the multiple, different editing events and variations in allele distribution might affect phenotypic outcome. Off-target effects of the Crispr/Cas9 reagents and other non-specific effects should be considered as well. It is possible to predict other sites in the genome that the reagents may target (Liu et al. 2019) and so one could sequence these sites to assess for off-target effects but this would not categorically rule out unexpected events at other sites in the genome.

Crispr/Cas9 genome editing in F0-injected fish can be a quick, economical way to work through a list of candidate genes to contribute eye phenotypes when disrupted and screen for potential loss of function phenotypes; however for validation and more complete investigation of phenotypes, it is advisable to establish lines carrying loss of function (or other) alleles. As we discuss below, the F0 approach also lends itself very effectively to screening for epistatic interactions and phenotypes dependent upon disruption of more than one gene function.

The first widely used approach for targeted abrogation of gene function was the injection of morpholino anti-sense oligonucleotides to prevent translation or disrupt splicing of the targeted gene (Heasman 2002; Nasevicius and Ekker 2000). Validated morpholinos are excellent reagents that provide a simple way to disrupt gene function. However, morpholinos can have off-target and non-specific effects and, consequently, rigorous controls are needed to be sure that an observed phenotype is due solely to the loss of function of the targeted gene (Blum et al. 2015; Eisen and Smith 2008; Stainier et al. 2017). Given that genome editing is now usually quite straightforward, it is advisable to establish stable lines of fish carrying loss of function alleles to facilitate validation of the efficacy of the morpholino. In zebrafish, it is currently much more challenging to create mutant alleles that disrupt splicing in a predictable way and consequently harder to robustly validate the phenotypic consequences of splice-blocking morpholinos. Accepting this caveat, splice-blocking morpholinos can be effective and useful if used judiciously with results described with appropriate caution.

The toolkit for genetic analyses of known or candidate human eye disease genes has improved tremendously in recent years and while no single approach is without limitations, together they enable investigators to both screen through many candidate genes quickly and pursue in-depth phenotypic analyses for those genes with phenotypes of greatest interest. Of course, there is no simple formula for the best experimental approach and experimental design should be tailored to the specific goals of the project. One of the most challenging future areas of research in this field is to understand how disruptions to functions of more than one gene can contribute to penetrance and expressivity of eye formation phenotypes and we finish our commentary by discussing how studies in fish will be able to advance this field of study.

## Modelling human eye disease in the zebrafish—what next?

The approaches described above allow us to target in zebrafish orthologues of genes associated with ocular malformations identified in humans. The disease models developed in this way have been instrumental to unravel the function of those genes, to identify the affected developmental processes and to start to understand pathology progression. In addition, the design of unbiased mutagenesis screens to identify mutations leading to eye defects in the zebrafish has resulted in the characterisation of novel genes important for eye formation, but not previously associated with ocular malformations in humans (Young et al. 2019). Despite these advances, the genes characterised to date constitute only the tip of the iceberg—in most cases, the genetic basis of the reported cases of ocular malformations in humans have not been identified (Plaisancie et al. 2019; Reis and Semina 2015; Williamson and FitzPatrick 2014). It is likely that in the near future, this list of candidate genes will keep expanding thanks to the extensive genetic data coming from projects such as UK Biobank and the Genomics England 100 K genome project (Turnbull et al. 2018). These new candidate disease genes will need to be validated and functionally characterised, and disrupting gene function in the zebrafish will enable fast progress in attaining this goal.

Still, it is likely that many of the reported cases of ocular malformations result not from defects in individual genes but from a combination of risk factors—mutations in multiple genes that in isolation have low penetrance/expressivity or do not result in a disease phenotype. Indeed, we now know that the existence of viable mutants in the population (in all species studied) is higher than previously suspected (Saleheen et al. 2017; Sulem et al. 2015). Despite not leading to overt phenotypes by themselves, these mutations with no effects on viability may constitute risk factors, sensitising the system to further mutations.

While many genes have been identified that participate in the early stages of eye specification and optic primordia formation, few of them lead to complete loss of eyes. Moreover, eye phenotypes in those mutant conditions in which there is an effect are often very variable, even between left and right eyes of one individual. This is true not only in model systems (Miesfeld et al. 2015; Young et al. 2019), but also in humans (Plaisancie et al. 2018; Reis and Semina 2015; Williamson et al. 2014). As mentioned above, a loss of function mutation may not have a phenotypic consequence due to redundancy, robustness in molecular pathways or developmental processes and genetic compensation. All these mechanisms may contribute to make eye morphogenesis a very robust process; however, when more than one mutation is present in an individual, the ability of the system to maintain homeostasis may start to falter, leading to the appearance of novel phenotypes.

A recent study of zebrafish carrying a null mutation in *tfc7l1a* nicely illustrates the dramatic consequences of combining mutations that by themselves do not result in disease phenotypes (Young et al. 2019). *tfc7l1a* homozygous mutant embryos are viable and develop normal eyes; however, the eye field of these mutants is much smaller than the wild type eye field. Despite this, the eye recovers its normal size by the end of embryogenesis, by prolonging the proliferative stage and delaying retinal differentiation. These mutants constitute a sensitised genetic background in which eye phenotypes manifest in the presence of additional mutations. Indeed, a synthetic enhancer screen performed in the *tfc7l1a* background led to the identification of three other genes that, when mutated in combination with *tfc7l1a*, lead to a strong reduction or complete loss of the eye primordium. *tfc7l1a* had also been found in a previous study to synergise with *tfap2a*, another gene involved in eye morphogenesis and associated with ocular malformations in humans (Gestri et al. 2009).

The robustness in eye formation revealed by the analysis of *tfc7l1a* mutant embryos suggests that the manifestation of MAC phenotypes in humans may also result from the combination of mutations in several genes. One potential limitation in identifying such combinatorial conditions lies in anticipating in which loss of function mutations actually sensitise the system to further mutations. The possibility of disrupting genes involved in eye formation by targeted mutagenesis will result in the establishment of many mutants that may provide sensitised conditions for disease gene discovery, such as the *tfc7l1a* mutant described above (Young et al. 2019). For instance, mutations in the *yap* gene lead to variably penetrant coloboma in both humans and zebrafish (Miesfeld et al. 2015; Williamson et al. 2014). The *yap*^*n113*^ zebrafish mutation is particularly useful as at normal temperature it shows unilateral coloboma only in about 15% of mutant embryos whereas penetrance increased and phenotypes become bilateral at higher temperature or when additional gene functions are disrupted (Miesfeld et al. 2015); Gestri and Wilson unpublished data). By targeting additional candidate disease genes with the F0 Crispr/Cas9 approach described above in these and other lines, we will be able to decipher the epistatic interactions and consequences of simultaneous disruption of two or more candidate MAC genes. We expect this to lead to the identification of many MAC genes that would otherwise be much more challenging to find using “one-gene-at-a-time” approaches. More traditional synthetic enhancer screens in sensitised mutant backgrounds—such as the one performed in the *tfc7l1a* background—will expand even further our list of candidate MAC genes. In addition, they will allow us to better understand the developmental pathways important for eye formation and will shed new insights into why there is such variability in MAC phenotypes. We anticipate many more years of fruitful research ahead in which the zebrafish will be a major player in this discovery process.
